# Pulmonary fat embolism: a potentially new fatal complication of SARS-CoV-2 infection. A case report

**DOI:** 10.1186/s12879-023-08559-4

**Published:** 2023-09-04

**Authors:** Jiangwei Kong, Tengfei Yang, Fu Zhang, XinBiao Liao, Sihao Du, Xingan Yang, Dongri Li

**Affiliations:** 1 Department of Forensic Pathology, School of Forensic Medicine, Southern Medical University, Guangzhou, Guangdong 510515 People’s Republic of China; 2 Forensic Pathology Lab, Guangdong Public Security Department, Forensic Department of Criminal Investigation Bureau, Guangzhou, Guangdong 510050 People’s Republic of China

**Keywords:** SARS-CoV-2, Pulmonary fat embolism, Pulmonary microthromboembolism, C-reactive protein

## Abstract

**Background:**

So far, there have been more than 761 million confirmed cases of SARS-CoV-2 worldwide, with more than 6.8 million deaths. The most common direct causes of death for COVID-19 are diffuse alveolar injury and acute respiratory distress syndrome. Autopsy results have shown that 80-100% of COVID-19 patients have microthrombi which is 9 times higher than in patients with influenza. There are reported cases of fat embolism associated with Covid-19, but relevant epidemiological investigations and fatal cases of pulmonary fat embolism are lacking. In this report, we describe the first COVID-19 patient to die from pulmonary fat embolism.

**Case presentation:**

A 54-year-old woman suddenly felt unwell while at work. She had difficulty breathing for 40 min and lost consciousness for 20 min before being taken to the hospital. On admission, her temperature was 36 ℃, but her respiration, heart rate, and blood pressure were undetectable. Laboratory examination revealed C-reactive protein, 26.55 mg/L; D-dimer, 11,400 µg/L; and procalcitonin, 0.21 ng/mL. She was declared clinically dead 2 h after admission due to ineffective rescue efforts. At autopsy, both lungs were highly oedematous with partial alveolar haemorrhage. The presence of microthrombi and pulmonary fat embolism in small interstitial pulmonary vessels was confirmed by phosphotungstic acid haematoxylin staining and oil red O staining. The immunohistochemical results of spike protein and nucleocapsid protein in laryngeal epithelial cells confirmed SARS-CoV-2 infection.

**Conclusions:**

Pulmonary fat embolism may be another fatal complication of COVID-19 infection, and clinicians should pay more attention to it.

**Supplementary Information:**

The online version contains supplementary material available at 10.1186/s12879-023-08559-4.

## Introduction

SARS-CoV-2 has rapidly spread around the world since it was first reported in December 2019. As of March 26, 2023, the World Health Organization reported more than 761 million confirmed cases and more than 6.8 million deaths [1]. Von Stillfried et al. [2]. reported 986 autopsy cases in a German multicentre study related to SARS-CoV-2 deaths, in which SARS-CoV-2 was the underlying cause of death in 86.2% of the cases. The most common direct causes of death are diffuse alveolar damage and acute respiratory distress syndrome. Only 4.0% of the deaths were due to pulmonary embolism. While fatal PFE was not mentioned in the study. Here, we report the first case of both fatal pulmonary microthromboembolism and PFE after infection with SARS-CoV-2. To our knowledge, there have been no reports of SARS-CoV-2-induced fatal PFE.

**Fig. 1:**
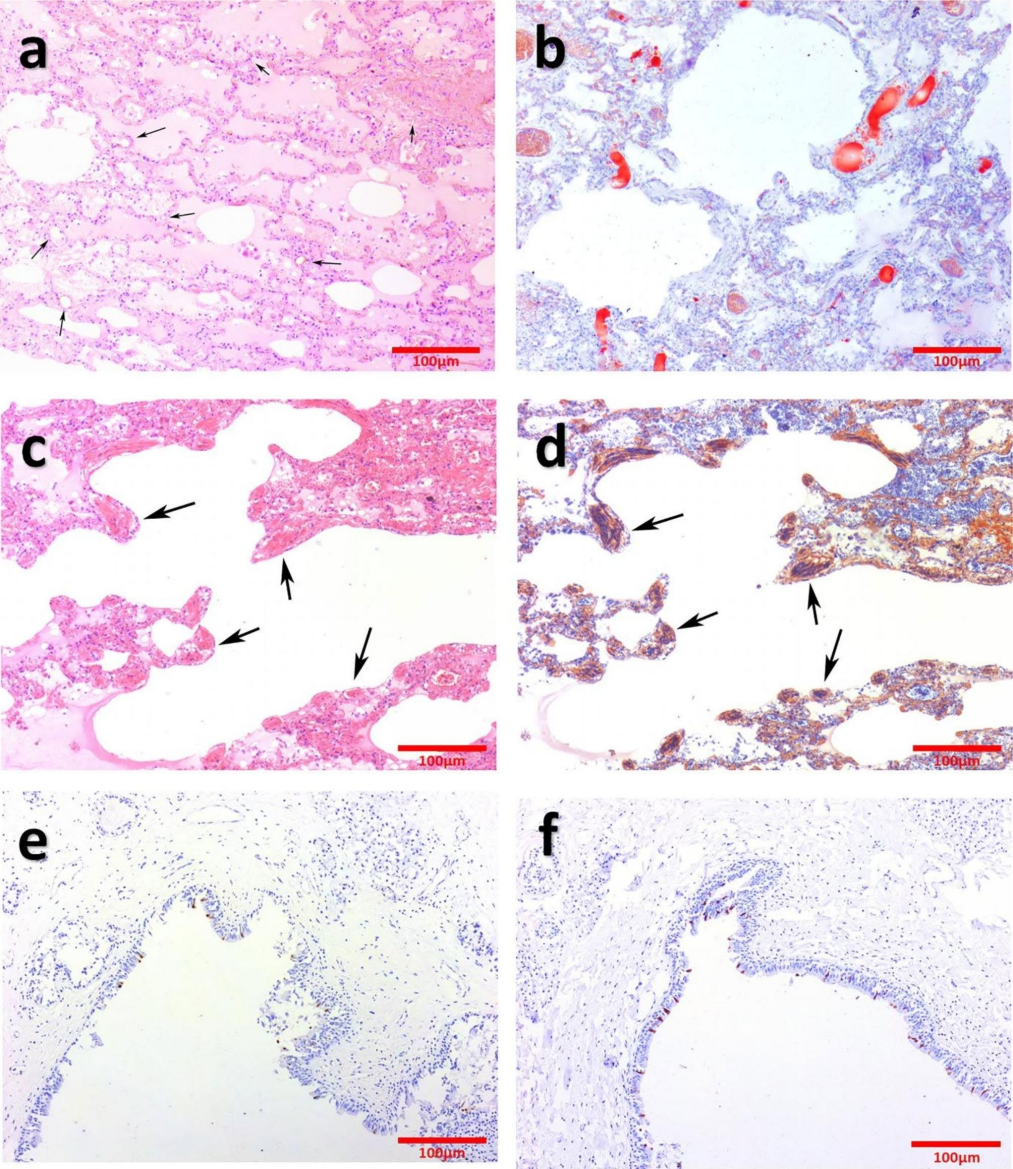
**a**: Diffuse pulmonary oedema, pulmonary haemorrhage and vacuole structure. (HE staining, arrows). **b**: Grade III fatal fat embolism in pulmonary interstitial small vessels. (ORO staining). c and **d**: Extensive microthrombus in pulmonary interstitial small vessels. (PTAH staining, arrows). **e** and **f**: Immunohistochemistry staining of S protein (E) and N protein (F)

### Case report

A 54-year-old woman suddenly felt unwell while at work. She had difficulty breathing for 40 min and lost consciousness for 20 min before being taken to the hospital. On admission, her temperature was 36 ℃, but her respiration, heart rate, and blood pressure were undetectable. She was pale orally and had cold limbs. Her bilateral pupils were dilated, fixed and were approximately 5 mm in diameter, and her light reflexes had disappeared. She lost carotid pulsation and was in asystole. Doctors immediately performed ardiopulmonary resuscitation, tracheal intubation, establishment of venous channels, heart strengthening and other rescue measures. Laboratory examination: CRP, 26.55 mg/L; D-dimer, 11,400 µg/L; PCT, 0.21 ng/mL. The patient died 2 h after admission. The clinician suspected that the cause of death was sudden cardiac death.

### External examination and autopsy

Forensic autopsy was performed 2 days after her death. External examination showed a well-nourished adult female, 145 cm in length, with no other fatal trauma other than rescue marks. A total of approximately 50 mL of light red fluid was seen in the bilateral thorax. Both lungs were congested and oedematous. The left lung weighed 600 g, and the right lung weighed 700 g. No lethal pathological changes were found in the other organs.

### Histopathology examination

Haematoxylin and eosin (HE) staining showed no abnormalities in the nervous and cardiovascular systems. Both lungs showed severe pulmonary oedema and pulmonary haemorrhage (Fig. 1a). There were suspected microthrombi and vacuolar structures in the lumen of extensive small pulmonary interstitial vessels. PTAH staining and ORO staining were performed, and the results showed the PFE (Fig. 1b) and microthrombi (Fig. 1c-d). Immunohistochemistry of the spike protein (Cat. No. 99423, Cell Signaling Technology, Danvers, MA) and nucleocapsid protein (Cat. No. 26369, Cell Signaling Technology, Danvers, MA) were performed on the larynx, right ventricle, adrenal gland and thymus. Only the laryngeal epithelial cells were positive (Fig. 1e-f), and the other organs were negative (Supplementary Fig. 1a-f).

## Discussion

Microthrombus embolism caused by SARS-CoV-2 has been a major threat of death. Ackermann et al. [3]. reported that the incidence of alveolar capillary microthrombi was 9 times higher in patients with COVID-19 than in patients with influenza. Autopsy results have shown that 80-100% of COVID-19 patients have microthrombi [4]. D-dimer is an important indicator indicating of thrombus formation. Fox et al. [5]. reported 10 autopsy cases of COVID-19 patients. Interestingly, six of these patients had significantly elevated D-dimers before death, and four were not tested. Their data suggested a correlation between elevated D-dimer levels and the outcome of fatal pulmonary embolism in COVID-19 patients. In this case, the patient is confirmed to be infected with SARS-CoV-2 by immunohistochemistry of the spike protein and nucleocapsids protein. The D-dimer level was much higher than the normal value, suggesting that the deceased was in a hypercoagulable state and most likely had thromboembolism. The results of HE and PTAH staining proved that she had microthrombus embolism. The current treatment guidelines related to COVID-19 worldwide mention the plan of anticoagulant therapy, which is the main reason why only 4% of the patients had pulmonary embolism cases occurred in the multicenter study of von Stillfried et al. [2].

Fat embolism is a pathological diagnosis of microvascular or capillary occlusion by lipid droplets in the lung or peripheral circulation. Since Zenker first described the presence of fat droplets in the lung of a railway worker who died of severe crush injury in the chest and abdomen in 1861, many scholars have explored the formation mechanism of fat embolism. The mechanical theory proposed by Guass [6] and the biochemical theory proposed by Lehman and Moore [7] have been accepted and applied by a large number of scholars.

On the basis of Lehman and Moore, Hulman et al. [8]. further confirmed the biochemical theory by conducting in vitro creaming tests on the serum of intensive care patients. Fat emboli are made up of chylomicron and very low density lipoprotein in the case of elevated plasma CRP and calcium dependency agglutination, forming a giant ball of fat with a diameter of 2–35 μm microns. CRP, an acute phase protein first described by Tillet and Francis, is a widely used biomarker of inflammation [9, 10]. Since early in the SARS-CoV-2 pandemic, high serum CRP concentrations, a good predictor of adverse outcome, have been associated with the severity and complications of COVID-19 patients [11]. To the best of our knowledge, there have been no reports of fatal PFE due to elevated CRP in patients with COVID-19. In this case, the CRP was as high as 26.55 mg/L before death, and the PCT was within the normal range, which could exclude the possibility of bacterial, fungal and parasitic infections. In the absence of other histopathological abnormalities, combined with the positive immunohistochemical results, it is reasonable to believe that the elevated CRP in this patient was caused by infection with SARS-CoV-2. According to the grading method of pulmonary fat embolism proposed by Falzi [12] and Sevitt [13], we evaluated the patient for grade III fatal PFE. Combined with the biochemical theory proposed by Lehman and Moore, we further believe that the patient was infected with SARS-CoV-2, which resulted in the formation of fatal PFE.

In addition to the widely accepted mechanical and biochemical theories, Cinti et al. [14]. proposed in 2020 that visceral fat necrosis and free fat droplet transfer into the vascular lumen in obese patients may be the key factors of SARS-CoV-2-induced pulmonary fat embolism. In 2022, Colleluori and Cinti et al. [15]. proved the authenticity of the conjecture by means of transmission electron microscopy and ORO staining. However, in this case, we did not find evidence of SARS-CoV-2 infection in the adipose tissue surrounding the right ventricle, adrenal gland and thymus (Supplementary Fig. 1a-f), so we cannot support this theory for the time being.

The clinical manifestations associated with fat embolism are known as fat embolism syndrome(FES). At present, the diagnostic criteria for FES mainly include Gurd’s criteria [16], Schonfeld’s criteria [17] and Lindeque’s criteria [18], but there is a lack of prospective studies with big data. In terms of laboratory diagnosis, in addition to the secondary criteria proposed by Gurd, IL-6 [19] and > 30% of macrophages in alveolar lavage fluid containing lipid droplets [20] are also considered as early diagnostic markers of FES. Imaging, as an auxiliary method, is characterized by interlobular septal thickening, ground glass opacity, diffuse nodules, and pulmonary consolidation [21]. However, these changes are still nonspecific. Yohsuke et al. [22]. detected fat in the right ventricle and pulmonary artery branches by computed tomography and magnetic resonance imaging of cadavers, providing an alternative method for the diagnosis of FE. In conclusion, a characteristic diagnosis of FES is still lacking and further studies are needed.

The treatment of FES is still at a supportive stage. Since the 1950s, people have tried a variety of drug treatments, including heparin, corticosteroids, albumin, aprotinin, hypertonic glucose, etc., but little effect has been achieved. The treatment of FES is still controversial and needs further research and exploration [23].

## Conclusions

We report the first case of microthromboembolism in small pulmonary vessels and fatal PFE after infection with SARS-CoV-2. In addition to common complications, we should also pay attention to the possibility of PFE, both in the current infection with SARS-CoV-2 and long COVID.

### Electronic supplementary material

Below is the link to the electronic supplementary material.

Supplementary Material 1: Fig 1. Immunohistochemistry in adipose tissue around the right ventricle, adrenal gland and thymus was negative. (Right ventricle: a, S protein; b, N protein. Adrenal gland: c, S protein; d, N protein. Thymus: e, S protein; f, N protein.)

## Data Availability

All data generated or analysed during this study are included in this published article [and its supplementary information files].

## Abbreviations

PFE: Pulmonary fat embolism
CRP: C-reactive protein
PCT: Procalcitonin
PTAH: Phosphotungstic acid haematoxylin
ORO: Oil red O
HE: Haematoxylin and eosin
FES: Fat embolism sydrome
