# Supplementary figures and images for: Pulmonary fat embolism: a potentially new fatal complication of SARS-CoV-2 infection. A case report
Source: BMC Infect Dis. 2023 Sep 4;23:576. doi: 10.1186/s12879-023-08559-4 (PMC10478277; doi:10.1186/s12879-023-08559-4)

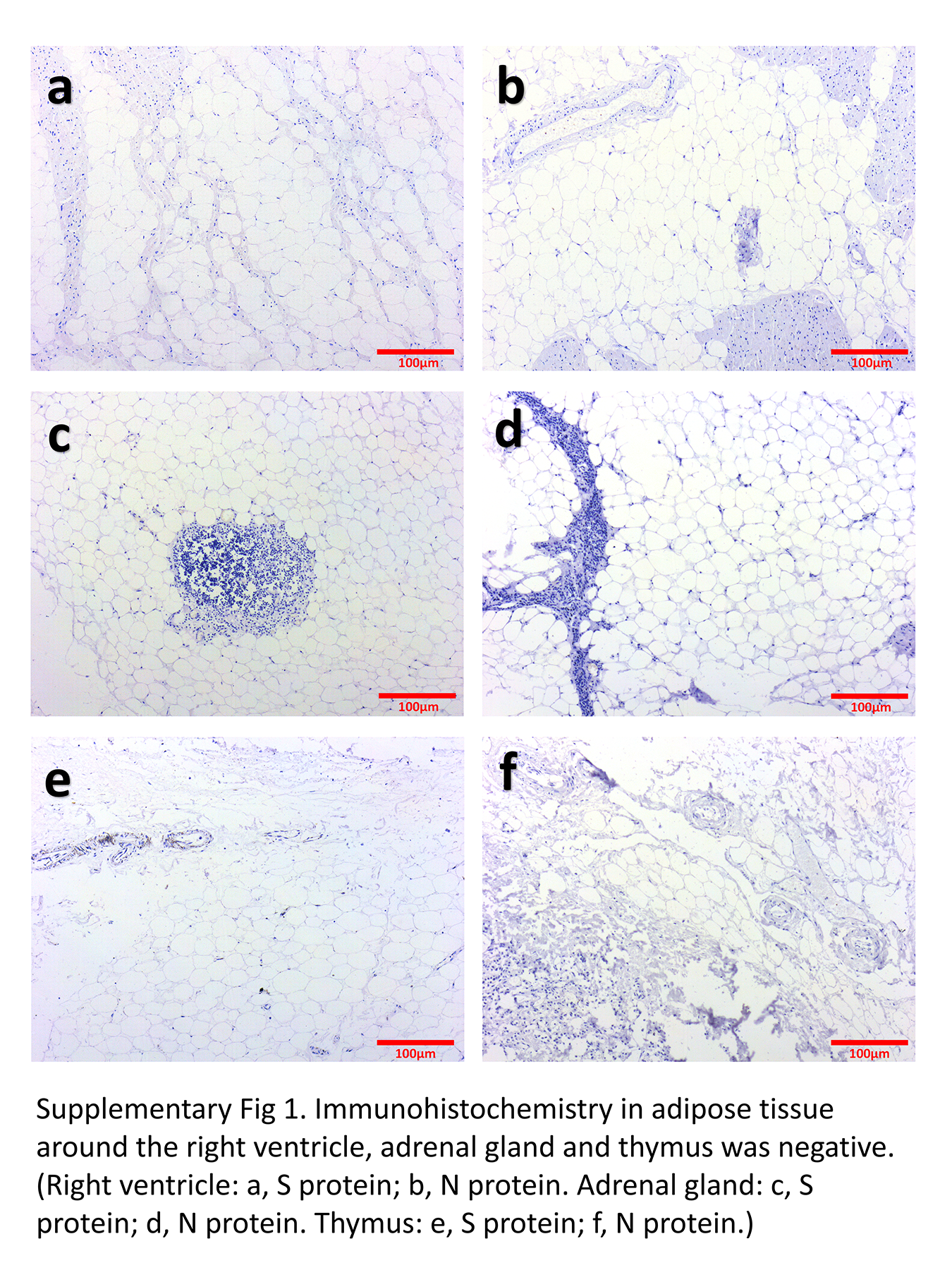

Supplement: Supplementary file 1 — Supplementary Material 1: Fig 1. Immunohistochemistry in adipose tissue around the right ventricle, adrenal gland and thymus was negative. (Right ventricle: a, S protein; b, N protein. Adrenal gland: c, S protein; d, N protein. Thymus: e, S protein; f, N protein.) [file 12879_2023_8559_MOESM1_ESM.png]
